# 1-[(2,3,4,5,6-Penta­fluoro­phen­yl)ethyn­yl]ferrocene

**DOI:** 10.1107/S1600536810026772

**Published:** 2010-07-10

**Authors:** Chan Zhang, Honglin Zhang, Dongdong Dai, Jinhua Liang

**Affiliations:** a Key Laboratory of Pesticides and Chemical Biology of the Ministry of Education, College of Chemistry, Central China Normal University, Wuhan 430079, People’s Republic of China

## Abstract

The mol­ecular structure of the title compound, [Fe(C_5_H_5_)(C_13_H_4_F_5_)], consists of a ferrocenyl group and a 2,3,4,5,6-penta­fluoro­benzene group linked through an ethyne spacer. The crystal packing is dominated by inter­molecular C—H⋯F hydrogen bonds, C—F⋯π inter­actions between the penta­fluoro­benzene groups [F⋯centroid distances = 3.882 (2) and 3.884 (2) Å] and π–π inter­actions between the penta­fluoro­benzene and cyclo­penta­dienyl rings [centroid–centroid distance = 3.741 (1) Å].

## Related literature

For general background to ferrocene derivatives, see: Debroy & Roy (2007 ▶). For a related structure, see: Valdebenito *et al.* (2010 ▶). For the synthesis, see: Torres *et al.* (2002 ▶); Zora *et al.* (2006 ▶).
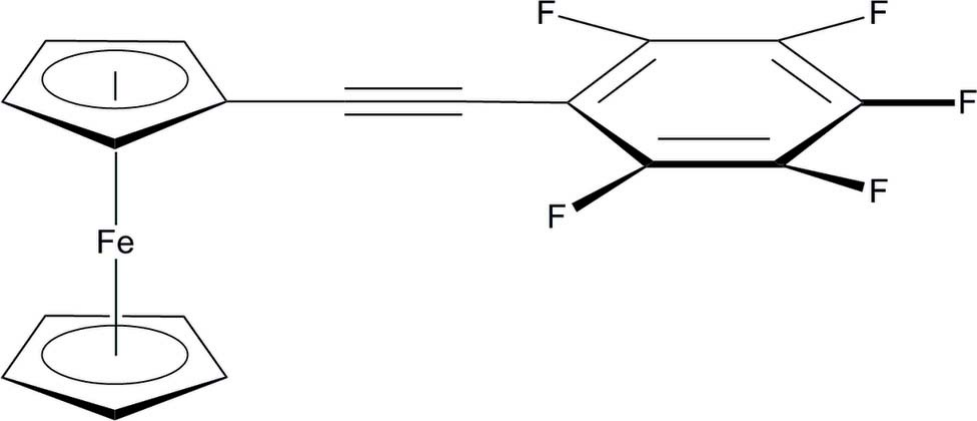


## Experimental

### 

#### Crystal data

[Fe(C_5_H_5_)(C_13_H_4_F_5_)]
*M* *_r_* = 376.10
Triclinic, 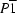

*a* = 6.0767 (9) Å
*b* = 10.7164 (15) Å
*c* = 12.6014 (18) Å
α = 65.725 (2)°
β = 89.364 (3)°
γ = 78.726 (2)°
*V* = 731.41 (18) Å^3^
*Z* = 2
Mo *K*α radiation
μ = 1.08 mm^−1^
*T* = 292 K
0.30 × 0.04 × 0.02 mm

#### Data collection

Bruker SMART APEX CCD diffractometer
4793 measured reflections
2533 independent reflections
2169 reflections with *I* > 2σ(*I*)
*R*_int_ = 0.021

#### Refinement

*R*[*F* ^2^ > 2σ(*F* ^2^)] = 0.040
*wR*(*F* ^2^) = 0.105
*S* = 1.05
2533 reflections
217 parameters
H-atom parameters constrained
Δρ_max_ = 0.41 e Å^−3^
Δρ_min_ = −0.25 e Å^−3^

### 

Data collection: *SMART* (Bruker, 2007 ▶); cell refinement: *SAINT* (Bruker, 2007 ▶); data reduction: *SAINT*; program(s) used to solve structure: *SHELXS97* (Sheldrick, 2008 ▶); program(s) used to refine structure: *SHELXL97* (Sheldrick, 2008 ▶); molecular graphics: *SHELXTL* (Sheldrick, 2008 ▶); software used to prepare material for publication: *SHELXTL*.

## Supplementary Material

Crystal structure: contains datablocks I, global. DOI: 10.1107/S1600536810026772/hy2327sup1.cif

Structure factors: contains datablocks I. DOI: 10.1107/S1600536810026772/hy2327Isup2.hkl

Additional supplementary materials:  crystallographic information; 3D view; checkCIF report

## Figures and Tables

**Table 1:** Hydrogen-bond geometry (Å, °)

*D*—H⋯*A*	*D*—H	H⋯*A*	*D*⋯*A*	*D*—H⋯*A*
C10—H10⋯F5^i^	0.93	2.50	3.176 (3)	130

Symmetry code: (i) 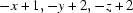.

**Fig. 1.**
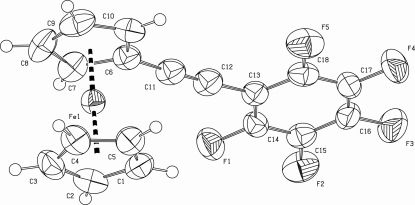
Molecular structure of the title compound. Displacement ellipsoids are drawn at the 50% probability level.

## supplementary crystallographic information

### Comment

Ferrocene derivatives with linear, unsaturated side-arms show altered unique properties, such as electrical conductivity, thermal stability, magnetic, electronic, redox and non-linear optical behaviors, compared to their saturated derivatives (Debroy & Roy, 2007).

The molecular structure of the title compound is composed of a ferrocenyl group and a pentafluorobenzene group joined by an organic ethyne spacer. Two cyclopentadienyl rings of the ferrocenyl group are parallel (Valdebenito *et al.*, 2010). The main features of the structure are an intermolecular C—H···F hydrogen bond between the ferrocenyl and pentafluorobenzene (Table 1), C—F···π interactions between the pentafluorobenzene groups [F···centroid distances = 3.882 (2) and 3.884 (2) Å] and a π–π interaction between the pentafluorobenzene and cyclopentadienyl rings [centroid–centroid distance = 3.741 (1) Å].

### Experimental

The title compound was synthesized according to a modified literature procedure (Torres *et al.*, 2002; Zora *et al.*, 2006). 1,2,3,4,5-Pentafluoro-6-iodobenzene (735 mg, 2.5 mmol) and ethynylferrocene (525 mg, 2.5 mmol) were added to a mixture of Cu(CH_3_CO_2_)_2_ (40 mg, 0.2 mmol), Pd(PPh_3_)_2_Cl_2_ (140 mg, 0.2 mmol) and ^i^Pr_2_NH (30 ml) under argon. The resulting mixture was refluxed at 365 K for 18 h. The solution was then extracted with dichloromethane, washed with water and dried over Na_2_SO_4_. Final purification was achieved by flash column chromatography on silica gel using dichloromethane/petroleum (1:4) as eluant. The product was obtained in 30% yield. Crystals of the title compound suitable for X-ray diffraction were obtained by slow evaporation of a solution of the compound in dichloromethane/hexane (1:10 v/v) at room temperature.

### Refinement

H atoms were positioned geometrically and refined as riding atoms, with C—H = 0.93 Å and with *U*_iso_(H) = 1.2*U*_eq_(C).

### Crystal data

**Table:**

[Fe(C_5_H_5_)(C_13_H_4_F_5_)]	*Z* = 2
*M**_r_* = 376.10	*F*(000) = 376
Triclinic, *P*1	*D*_x_ = 1.708 Mg m^−^^3^
Hall symbol: -P 1	Mo *K*α radiation, λ = 0.71073 Å
*a* = 6.0767 (9) Å	Cell parameters from 1760 reflections
*b* = 10.7164 (15) Å	θ = 3.3–25.1°
*c* = 12.6014 (18) Å	µ = 1.08 mm^−^^1^
α = 65.725 (2)°	*T* = 292 K
β = 89.364 (3)°	Plate, yellow
γ = 78.726 (2)°	0.30 × 0.04 × 0.02 mm
*V* = 731.41 (18) Å^3^	

### Data collection

**Table:**

Bruker SMART APEX CCD diffractometer	2169 reflections with *I* > 2σ(*I*)
Radiation source: fine-focus sealed tube	*R*_int_ = 0.021
graphite	θ_max_ = 25.0°, θ_min_ = 1.8°
φ and ω scans	*h* = −7→7
4793 measured reflections	*k* = −12→12
2533 independent reflections	*l* = −13→14

### Refinement

**Table:**

Refinement on *F*^2^	Primary atom site location: structure-invariant direct methods
Least-squares matrix: full	Secondary atom site location: difference Fourier map
*R*[*F*^2^ > 2σ(*F*^2^)] = 0.040	Hydrogen site location: inferred from neighbouring sites
*wR*(*F*^2^) = 0.105	H-atom parameters constrained
*S* = 1.05	*w* = 1/[σ^2^(*F*_o_^2^) + (0.0602*P*)^2^ + 0.0784*P*] where *P* = (*F*_o_^2^ + 2*F*_c_^2^)/3
2533 reflections	(Δ/σ)_max_ = 0.001
217 parameters	Δρ_max_ = 0.41 e Å^−^^3^
0 restraints	Δρ_min_ = −0.25 e Å^−^^3^

### Fractional atomic coordinates and isotropic or equivalent isotropic displacement parameters (Å^2^)

**Table:**

	*x*	*y*	*z*	*U*_iso_*/*U*_eq_	
Fe1	0.72848 (6)	0.78028 (4)	0.74835 (3)	0.03952 (17)	
C1	0.7513 (5)	0.5868 (3)	0.8801 (3)	0.0544 (8)	
H1	0.7967	0.5607	0.9578	0.065*	
C2	0.8930 (6)	0.5793 (3)	0.7932 (3)	0.0603 (9)	
H2	1.0483	0.5472	0.8031	0.072*	
C3	0.7575 (6)	0.6290 (3)	0.6885 (3)	0.0629 (9)	
H3	0.8079	0.6360	0.6167	0.075*	
C4	0.5318 (5)	0.6666 (3)	0.7110 (3)	0.0550 (8)	
H4	0.4074	0.7022	0.6570	0.066*	
C5	0.5292 (5)	0.6405 (3)	0.8295 (3)	0.0534 (8)	
H5	0.4024	0.6561	0.8679	0.064*	
C6	0.8628 (5)	0.8977 (3)	0.8131 (3)	0.0466 (7)	
C7	0.9602 (5)	0.9037 (3)	0.7080 (3)	0.0537 (8)	
H7	1.1120	0.8756	0.7001	0.064*	
C8	0.7850 (6)	0.9598 (3)	0.6182 (3)	0.0612 (9)	
H8	0.8017	0.9753	0.5406	0.073*	
C9	0.5823 (6)	0.9883 (3)	0.6656 (3)	0.0620 (9)	
H9	0.4417	1.0264	0.6245	0.074*	
C10	0.6256 (5)	0.9498 (3)	0.7858 (3)	0.0535 (8)	
H10	0.5195	0.9569	0.8379	0.064*	
C11	0.9780 (5)	0.8492 (3)	0.9247 (3)	0.0506 (7)	
C12	1.0717 (5)	0.8129 (3)	1.0184 (3)	0.0524 (7)	
C13	1.1670 (5)	0.7703 (3)	1.1340 (2)	0.0434 (6)	
C14	1.3917 (5)	0.7049 (3)	1.1689 (2)	0.0441 (7)	
C15	1.4796 (5)	0.6621 (3)	1.2811 (3)	0.0496 (7)	
C16	1.3452 (6)	0.6841 (3)	1.3614 (2)	0.0539 (8)	
C17	1.1218 (6)	0.7482 (3)	1.3302 (3)	0.0551 (8)	
C18	1.0362 (5)	0.7906 (3)	1.2187 (3)	0.0518 (8)	
F1	1.5272 (3)	0.68281 (19)	1.09132 (16)	0.0650 (5)	
F2	1.6961 (3)	0.59629 (19)	1.31194 (17)	0.0709 (5)	
F3	1.4306 (4)	0.6459 (2)	1.46997 (16)	0.0892 (7)	
F4	0.9884 (4)	0.7688 (2)	1.40989 (18)	0.0896 (7)	
F5	0.8196 (3)	0.85479 (19)	1.19000 (19)	0.0741 (6)	

### Atomic displacement parameters (Å^2^)

**Table:**

	*U*^11^	*U*^22^	*U*^33^	*U*^12^	*U*^13^	*U*^23^
Fe1	0.0398 (3)	0.0418 (3)	0.0403 (3)	−0.01159 (17)	0.00257 (18)	−0.01908 (19)
C1	0.067 (2)	0.0416 (15)	0.0485 (18)	−0.0124 (14)	−0.0050 (16)	−0.0118 (14)
C2	0.0542 (19)	0.0474 (17)	0.085 (3)	−0.0053 (14)	0.0022 (18)	−0.0358 (17)
C3	0.085 (2)	0.0606 (19)	0.065 (2)	−0.0260 (17)	0.0217 (19)	−0.0425 (18)
C4	0.0571 (19)	0.0602 (18)	0.060 (2)	−0.0209 (15)	−0.0019 (16)	−0.0336 (16)
C5	0.0535 (19)	0.0519 (17)	0.059 (2)	−0.0222 (14)	0.0127 (16)	−0.0228 (15)
C6	0.0520 (18)	0.0408 (14)	0.0502 (17)	−0.0126 (12)	−0.0047 (14)	−0.0207 (13)
C7	0.0466 (17)	0.0569 (17)	0.057 (2)	−0.0210 (14)	0.0025 (15)	−0.0188 (15)
C8	0.069 (2)	0.0617 (19)	0.0443 (18)	−0.0308 (17)	−0.0013 (16)	−0.0060 (15)
C9	0.056 (2)	0.0463 (17)	0.071 (2)	−0.0078 (14)	−0.0208 (17)	−0.0127 (16)
C10	0.0512 (18)	0.0439 (15)	0.067 (2)	−0.0031 (13)	−0.0014 (15)	−0.0278 (15)
C11	0.0554 (19)	0.0433 (15)	0.0563 (19)	−0.0155 (13)	−0.0044 (15)	−0.0215 (14)
C12	0.0585 (19)	0.0470 (16)	0.055 (2)	−0.0173 (14)	−0.0031 (16)	−0.0210 (15)
C13	0.0499 (17)	0.0384 (14)	0.0460 (16)	−0.0157 (12)	0.0008 (13)	−0.0188 (13)
C14	0.0471 (17)	0.0444 (14)	0.0464 (17)	−0.0138 (12)	0.0086 (14)	−0.0228 (13)
C15	0.0514 (18)	0.0406 (15)	0.0554 (19)	−0.0124 (13)	−0.0034 (15)	−0.0173 (14)
C16	0.085 (2)	0.0417 (15)	0.0356 (16)	−0.0192 (15)	−0.0014 (16)	−0.0136 (13)
C17	0.080 (2)	0.0444 (16)	0.0496 (19)	−0.0222 (15)	0.0240 (17)	−0.0240 (14)
C18	0.0484 (18)	0.0395 (15)	0.068 (2)	−0.0123 (13)	0.0092 (16)	−0.0218 (15)
F1	0.0653 (12)	0.0744 (11)	0.0630 (11)	−0.0143 (9)	0.0201 (9)	−0.0366 (10)
F2	0.0557 (12)	0.0658 (11)	0.0808 (13)	−0.0075 (9)	−0.0152 (10)	−0.0222 (10)
F3	0.147 (2)	0.0710 (12)	0.0463 (11)	−0.0216 (13)	−0.0146 (12)	−0.0211 (10)
F4	0.1264 (19)	0.0795 (13)	0.0750 (13)	−0.0281 (12)	0.0557 (13)	−0.0424 (11)
F5	0.0497 (11)	0.0640 (11)	0.1014 (15)	−0.0069 (9)	0.0147 (10)	−0.0299 (11)

### Geometric parameters (Å, °)

**Table:**

Fe1—C3	2.029 (3)	C6—C10	1.430 (4)
Fe1—C2	2.032 (3)	C7—C8	1.411 (4)
Fe1—C1	2.034 (3)	C7—H7	0.9300
Fe1—C7	2.037 (3)	C8—C9	1.398 (4)
Fe1—C8	2.040 (3)	C8—H8	0.9300
Fe1—C10	2.042 (3)	C9—C10	1.409 (4)
Fe1—C5	2.044 (3)	C9—H9	0.9300
Fe1—C6	2.044 (3)	C10—H10	0.9300
Fe1—C9	2.045 (3)	C11—C12	1.191 (4)
Fe1—C4	2.047 (3)	C12—C13	1.427 (4)
C1—C2	1.404 (4)	C13—C18	1.386 (4)
C1—C5	1.405 (4)	C13—C14	1.391 (4)
C1—H1	0.9300	C14—F1	1.336 (3)
C2—C3	1.406 (5)	C14—C15	1.372 (4)
C2—H2	0.9300	C15—F2	1.342 (3)
C3—C4	1.412 (4)	C15—C16	1.360 (4)
C3—H3	0.9300	C16—F3	1.335 (3)
C4—C5	1.402 (4)	C16—C17	1.376 (5)
C4—H4	0.9300	C17—F4	1.345 (3)
C5—H5	0.9300	C17—C18	1.360 (4)
C6—C11	1.421 (4)	C18—F5	1.337 (3)
C6—C7	1.426 (4)		
			
C3—Fe1—C2	40.51 (13)	C5—C4—C3	107.6 (3)
C3—Fe1—C1	67.85 (13)	C5—C4—Fe1	69.83 (16)
C2—Fe1—C1	40.41 (12)	C3—C4—Fe1	69.08 (17)
C3—Fe1—C7	118.88 (13)	C5—C4—H4	126.2
C2—Fe1—C7	108.34 (13)	C3—C4—H4	126.2
C1—Fe1—C7	128.23 (13)	Fe1—C4—H4	126.4
C3—Fe1—C8	109.38 (14)	C4—C5—C1	108.2 (3)
C2—Fe1—C8	129.09 (14)	C4—C5—Fe1	70.07 (16)
C1—Fe1—C8	166.63 (13)	C1—C5—Fe1	69.47 (16)
C7—Fe1—C8	40.51 (12)	C4—C5—H5	125.9
C3—Fe1—C10	166.11 (14)	C1—C5—H5	125.9
C2—Fe1—C10	151.56 (14)	Fe1—C5—H5	126.1
C1—Fe1—C10	117.85 (13)	C11—C6—C7	126.8 (3)
C7—Fe1—C10	68.73 (12)	C11—C6—C10	125.8 (3)
C8—Fe1—C10	68.07 (14)	C7—C6—C10	107.4 (3)
C3—Fe1—C5	67.75 (13)	C11—C6—Fe1	126.48 (19)
C2—Fe1—C5	67.92 (13)	C7—C6—Fe1	69.29 (15)
C1—Fe1—C5	40.30 (12)	C10—C6—Fe1	69.44 (16)
C7—Fe1—C5	165.96 (12)	C8—C7—C6	107.7 (3)
C8—Fe1—C5	152.15 (13)	C8—C7—Fe1	69.85 (17)
C10—Fe1—C5	107.70 (13)	C6—C7—Fe1	69.79 (16)
C3—Fe1—C6	152.12 (14)	C8—C7—H7	126.1
C2—Fe1—C6	117.98 (13)	C6—C7—H7	126.1
C1—Fe1—C6	107.64 (12)	Fe1—C7—H7	125.8
C7—Fe1—C6	40.92 (11)	C9—C8—C7	108.5 (3)
C8—Fe1—C6	68.29 (13)	C9—C8—Fe1	70.17 (17)
C10—Fe1—C6	40.98 (11)	C7—C8—Fe1	69.64 (17)
C5—Fe1—C6	127.66 (12)	C9—C8—H8	125.8
C3—Fe1—C9	129.10 (14)	C7—C8—H8	125.8
C2—Fe1—C9	166.84 (14)	Fe1—C8—H8	126.0
C1—Fe1—C9	151.75 (14)	C8—C9—C10	108.9 (3)
C7—Fe1—C9	67.90 (13)	C8—C9—Fe1	69.80 (18)
C8—Fe1—C9	40.03 (13)	C10—C9—Fe1	69.72 (16)
C10—Fe1—C9	40.35 (12)	C8—C9—H9	125.5
C5—Fe1—C9	118.81 (13)	C10—C9—H9	125.5
C6—Fe1—C9	68.10 (12)	Fe1—C9—H9	126.5
C3—Fe1—C4	40.53 (13)	C9—C10—C6	107.4 (3)
C2—Fe1—C4	68.12 (13)	C9—C10—Fe1	69.93 (17)
C1—Fe1—C4	67.74 (13)	C6—C10—Fe1	69.58 (15)
C7—Fe1—C4	152.61 (13)	C9—C10—H10	126.3
C8—Fe1—C4	119.34 (13)	C6—C10—H10	126.3
C10—Fe1—C4	127.70 (12)	Fe1—C10—H10	125.8
C5—Fe1—C4	40.10 (12)	C12—C11—C6	177.7 (3)
C6—Fe1—C4	165.50 (12)	C11—C12—C13	175.5 (3)
C9—Fe1—C4	109.02 (13)	C18—C13—C14	116.4 (3)
C2—C1—C5	108.3 (3)	C18—C13—C12	120.9 (3)
C2—C1—Fe1	69.73 (17)	C14—C13—C12	122.7 (3)
C5—C1—Fe1	70.23 (16)	F1—C14—C15	118.6 (3)
C2—C1—H1	125.8	F1—C14—C13	119.4 (3)
C5—C1—H1	125.8	C15—C14—C13	122.0 (3)
Fe1—C1—H1	125.8	F2—C15—C16	120.2 (3)
C1—C2—C3	107.6 (3)	F2—C15—C14	120.1 (3)
C1—C2—Fe1	69.86 (16)	C16—C15—C14	119.6 (3)
C3—C2—Fe1	69.65 (17)	F3—C16—C15	120.0 (3)
C1—C2—H2	126.2	F3—C16—C17	119.9 (3)
C3—C2—H2	126.2	C15—C16—C17	120.0 (3)
Fe1—C2—H2	125.9	F4—C17—C18	120.1 (3)
C2—C3—C4	108.3 (3)	F4—C17—C16	120.0 (3)
C2—C3—Fe1	69.84 (17)	C18—C17—C16	119.9 (3)
C4—C3—Fe1	70.39 (17)	F5—C18—C17	118.8 (3)
C2—C3—H3	125.8	F5—C18—C13	119.2 (3)
C4—C3—H3	125.8	C17—C18—C13	122.0 (3)
Fe1—C3—H3	125.5		
			
C3—Fe1—C1—C2	37.99 (19)	C1—Fe1—C6—C10	−112.43 (19)
C7—Fe1—C1—C2	−72.1 (2)	C7—Fe1—C6—C10	118.9 (3)
C8—Fe1—C1—C2	−42.6 (6)	C8—Fe1—C6—C10	81.1 (2)
C10—Fe1—C1—C2	−155.99 (19)	C5—Fe1—C6—C10	−72.4 (2)
C5—Fe1—C1—C2	119.2 (3)	C9—Fe1—C6—C10	37.84 (18)
C6—Fe1—C1—C2	−112.7 (2)	C4—Fe1—C6—C10	−43.4 (5)
C9—Fe1—C1—C2	170.9 (2)	C11—C6—C7—C8	−179.5 (3)
C4—Fe1—C1—C2	81.9 (2)	C10—C6—C7—C8	0.6 (3)
C3—Fe1—C1—C5	−81.3 (2)	Fe1—C6—C7—C8	59.8 (2)
C2—Fe1—C1—C5	−119.2 (3)	C11—C6—C7—Fe1	120.7 (3)
C7—Fe1—C1—C5	168.67 (17)	C10—C6—C7—Fe1	−59.20 (19)
C8—Fe1—C1—C5	−161.9 (5)	C3—Fe1—C7—C8	86.4 (2)
C10—Fe1—C1—C5	84.8 (2)	C2—Fe1—C7—C8	129.4 (2)
C6—Fe1—C1—C5	128.05 (18)	C1—Fe1—C7—C8	169.92 (19)
C9—Fe1—C1—C5	51.7 (3)	C10—Fe1—C7—C8	−80.7 (2)
C4—Fe1—C1—C5	−37.32 (18)	C5—Fe1—C7—C8	−158.5 (4)
C5—C1—C2—C3	0.2 (3)	C6—Fe1—C7—C8	−118.8 (3)
Fe1—C1—C2—C3	−59.7 (2)	C9—Fe1—C7—C8	−37.2 (2)
C5—C1—C2—Fe1	59.9 (2)	C4—Fe1—C7—C8	51.7 (4)
C3—Fe1—C2—C1	−118.6 (3)	C3—Fe1—C7—C6	−154.81 (18)
C7—Fe1—C2—C1	128.06 (19)	C2—Fe1—C7—C6	−111.85 (19)
C8—Fe1—C2—C1	168.36 (18)	C1—Fe1—C7—C6	−71.3 (2)
C10—Fe1—C2—C1	49.0 (3)	C8—Fe1—C7—C6	118.8 (3)
C5—Fe1—C2—C1	−37.52 (18)	C10—Fe1—C7—C6	38.03 (17)
C6—Fe1—C2—C1	84.6 (2)	C5—Fe1—C7—C6	−39.7 (6)
C9—Fe1—C2—C1	−160.8 (5)	C9—Fe1—C7—C6	81.60 (19)
C4—Fe1—C2—C1	−80.9 (2)	C4—Fe1—C7—C6	170.5 (2)
C1—Fe1—C2—C3	118.6 (3)	C6—C7—C8—C9	−0.1 (3)
C7—Fe1—C2—C3	−113.3 (2)	Fe1—C7—C8—C9	59.6 (2)
C8—Fe1—C2—C3	−73.0 (2)	C6—C7—C8—Fe1	−59.75 (19)
C10—Fe1—C2—C3	167.7 (2)	C3—Fe1—C8—C9	128.4 (2)
C5—Fe1—C2—C3	81.1 (2)	C2—Fe1—C8—C9	169.54 (19)
C6—Fe1—C2—C3	−156.79 (19)	C1—Fe1—C8—C9	−156.0 (5)
C9—Fe1—C2—C3	−42.2 (6)	C7—Fe1—C8—C9	−119.5 (3)
C4—Fe1—C2—C3	37.74 (18)	C10—Fe1—C8—C9	−37.05 (19)
C1—C2—C3—C4	−0.3 (3)	C5—Fe1—C8—C9	49.5 (4)
Fe1—C2—C3—C4	−60.1 (2)	C6—Fe1—C8—C9	−81.4 (2)
C1—C2—C3—Fe1	59.8 (2)	C4—Fe1—C8—C9	85.0 (2)
C1—Fe1—C3—C2	−37.89 (19)	C3—Fe1—C8—C7	−112.1 (2)
C7—Fe1—C3—C2	84.7 (2)	C2—Fe1—C8—C7	−70.9 (2)
C8—Fe1—C3—C2	128.1 (2)	C1—Fe1—C8—C7	−36.5 (6)
C10—Fe1—C3—C2	−155.0 (5)	C10—Fe1—C8—C7	82.5 (2)
C5—Fe1—C3—C2	−81.6 (2)	C5—Fe1—C8—C7	169.0 (2)
C6—Fe1—C3—C2	48.1 (3)	C6—Fe1—C8—C7	38.17 (19)
C9—Fe1—C3—C2	168.64 (19)	C9—Fe1—C8—C7	119.5 (3)
C4—Fe1—C3—C2	−119.1 (3)	C4—Fe1—C8—C7	−155.52 (19)
C2—Fe1—C3—C4	119.1 (3)	C7—C8—C9—C10	−0.4 (4)
C1—Fe1—C3—C4	81.2 (2)	Fe1—C8—C9—C10	58.9 (2)
C7—Fe1—C3—C4	−156.23 (18)	C7—C8—C9—Fe1	−59.3 (2)
C8—Fe1—C3—C4	−112.8 (2)	C3—Fe1—C9—C8	−72.4 (2)
C10—Fe1—C3—C4	−35.9 (6)	C2—Fe1—C9—C8	−38.2 (6)
C5—Fe1—C3—C4	37.50 (18)	C1—Fe1—C9—C8	168.5 (2)
C6—Fe1—C3—C4	167.2 (2)	C7—Fe1—C9—C8	37.59 (19)
C9—Fe1—C3—C4	−72.3 (2)	C10—Fe1—C9—C8	120.3 (3)
C2—C3—C4—C5	0.3 (3)	C5—Fe1—C9—C8	−156.08 (19)
Fe1—C3—C4—C5	−59.5 (2)	C6—Fe1—C9—C8	81.9 (2)
C2—C3—C4—Fe1	59.8 (2)	C4—Fe1—C9—C8	−113.3 (2)
C3—Fe1—C4—C5	119.0 (3)	C3—Fe1—C9—C10	167.30 (18)
C2—Fe1—C4—C5	81.3 (2)	C2—Fe1—C9—C10	−158.5 (5)
C1—Fe1—C4—C5	37.50 (18)	C1—Fe1—C9—C10	48.2 (3)
C7—Fe1—C4—C5	169.1 (2)	C7—Fe1—C9—C10	−82.72 (19)
C8—Fe1—C4—C5	−155.12 (19)	C8—Fe1—C9—C10	−120.3 (3)
C10—Fe1—C4—C5	−71.3 (2)	C5—Fe1—C9—C10	83.6 (2)
C6—Fe1—C4—C5	−36.5 (5)	C6—Fe1—C9—C10	−38.43 (18)
C9—Fe1—C4—C5	−112.5 (2)	C4—Fe1—C9—C10	126.40 (19)
C2—Fe1—C4—C3	−37.72 (19)	C8—C9—C10—C6	0.8 (3)
C1—Fe1—C4—C3	−81.5 (2)	Fe1—C9—C10—C6	59.73 (19)
C7—Fe1—C4—C3	50.1 (4)	C8—C9—C10—Fe1	−58.9 (2)
C8—Fe1—C4—C3	85.9 (2)	C11—C6—C10—C9	179.2 (3)
C10—Fe1—C4—C3	169.76 (19)	C7—C6—C10—C9	−0.8 (3)
C5—Fe1—C4—C3	−119.0 (3)	Fe1—C6—C10—C9	−60.0 (2)
C6—Fe1—C4—C3	−155.5 (4)	C11—C6—C10—Fe1	−120.8 (3)
C9—Fe1—C4—C3	128.6 (2)	C7—C6—C10—Fe1	59.11 (19)
C3—C4—C5—C1	−0.2 (3)	C3—Fe1—C10—C9	−45.3 (6)
Fe1—C4—C5—C1	−59.2 (2)	C2—Fe1—C10—C9	169.9 (2)
C3—C4—C5—Fe1	59.0 (2)	C1—Fe1—C10—C9	−156.46 (19)
C2—C1—C5—C4	0.0 (3)	C7—Fe1—C10—C9	80.5 (2)
Fe1—C1—C5—C4	59.6 (2)	C8—Fe1—C10—C9	36.76 (19)
C2—C1—C5—Fe1	−59.6 (2)	C5—Fe1—C10—C9	−113.9 (2)
C3—Fe1—C5—C4	−37.89 (19)	C6—Fe1—C10—C9	118.5 (3)
C2—Fe1—C5—C4	−81.8 (2)	C4—Fe1—C10—C9	−74.1 (2)
C1—Fe1—C5—C4	−119.4 (3)	C3—Fe1—C10—C6	−163.8 (5)
C7—Fe1—C5—C4	−158.9 (5)	C2—Fe1—C10—C6	51.5 (3)
C8—Fe1—C5—C4	51.7 (3)	C1—Fe1—C10—C6	85.1 (2)
C10—Fe1—C5—C4	128.14 (19)	C7—Fe1—C10—C6	−37.97 (17)
C6—Fe1—C5—C4	169.15 (17)	C8—Fe1—C10—C6	−81.69 (19)
C9—Fe1—C5—C4	85.7 (2)	C5—Fe1—C10—C6	127.62 (18)
C3—Fe1—C5—C1	81.5 (2)	C9—Fe1—C10—C6	−118.5 (3)
C2—Fe1—C5—C1	37.61 (19)	C4—Fe1—C10—C6	167.43 (17)
C7—Fe1—C5—C1	−39.5 (6)	C18—C13—C14—F1	−179.7 (2)
C8—Fe1—C5—C1	171.1 (3)	C12—C13—C14—F1	1.4 (4)
C10—Fe1—C5—C1	−112.45 (19)	C18—C13—C14—C15	0.2 (4)
C6—Fe1—C5—C1	−71.4 (2)	C12—C13—C14—C15	−178.7 (3)
C9—Fe1—C5—C1	−154.93 (19)	F1—C14—C15—F2	−1.7 (4)
C4—Fe1—C5—C1	119.4 (3)	C13—C14—C15—F2	178.4 (2)
C3—Fe1—C6—C11	−68.3 (4)	F1—C14—C15—C16	179.7 (2)
C2—Fe1—C6—C11	−35.1 (3)	C13—C14—C15—C16	−0.2 (4)
C1—Fe1—C6—C11	7.5 (3)	F2—C15—C16—F3	3.0 (4)
C7—Fe1—C6—C11	−121.1 (3)	C14—C15—C16—F3	−178.4 (2)
C8—Fe1—C6—C11	−158.9 (3)	F2—C15—C16—C17	−178.2 (2)
C10—Fe1—C6—C11	120.0 (3)	C14—C15—C16—C17	0.4 (4)
C5—Fe1—C6—C11	47.6 (3)	F3—C16—C17—F4	−1.9 (4)
C9—Fe1—C6—C11	157.8 (3)	C15—C16—C17—F4	179.4 (3)
C4—Fe1—C6—C11	76.5 (5)	F3—C16—C17—C18	178.2 (2)
C3—Fe1—C6—C7	52.8 (3)	C15—C16—C17—C18	−0.6 (4)
C2—Fe1—C6—C7	86.0 (2)	F4—C17—C18—F5	1.2 (4)
C1—Fe1—C6—C7	128.66 (18)	C16—C17—C18—F5	−178.9 (2)
C8—Fe1—C6—C7	−37.81 (19)	F4—C17—C18—C13	−179.3 (2)
C10—Fe1—C6—C7	−118.9 (3)	C16—C17—C18—C13	0.6 (4)
C5—Fe1—C6—C7	168.71 (17)	C14—C13—C18—F5	179.1 (2)
C9—Fe1—C6—C7	−81.1 (2)	C12—C13—C18—F5	−2.0 (4)
C4—Fe1—C6—C7	−162.3 (4)	C14—C13—C18—C17	−0.4 (4)
C3—Fe1—C6—C10	171.8 (2)	C12—C13—C18—C17	178.5 (3)
C2—Fe1—C6—C10	−155.05 (18)		

### Hydrogen-bond geometry (Å, °)

**Table:**

*D*—H···*A*	*D*—H	H···*A*	*D*···*A*	*D*—H···*A*
C10—H10···F5^i^	0.93	2.50	3.176 (3)	130

Symmetry codes: (i) −*x*+1, −*y*+2, −*z*+2.
